# MOSTPLAS: a self-correction multi-label learning model for plasmid host range prediction

**DOI:** 10.1093/bioinformatics/btaf075

**Published:** 2025-02-17

**Authors:** Wei Zou, Yongxin Ji, Jiaojiao Guan, Yanni Sun

**Affiliations:** Department of Electrical Engineering, City University of Hong Kong, 83 Tat Chee Avenue, Kowloon, Hong Kong SAR, China; Department of Electrical Engineering, City University of Hong Kong, 83 Tat Chee Avenue, Kowloon, Hong Kong SAR, China; Department of Electrical Engineering, City University of Hong Kong, 83 Tat Chee Avenue, Kowloon, Hong Kong SAR, China; Department of Electrical Engineering, City University of Hong Kong, 83 Tat Chee Avenue, Kowloon, Hong Kong SAR, China

## Abstract

**Motivation:**

Plasmids play an essential role in horizontal gene transfer, aiding their host bacteria in acquiring beneficial traits like antibiotic and metal resistance. There exist some plasmids that can transfer, replicate, or persist in multiple organisms. Identifying the relatively complete host range of these plasmids provides insights into how plasmids promote bacterial evolution. To achieve this, we can apply multi-label learning models for plasmid host range prediction. However, there are no databases providing the detailed and complete host labels of these broad-host-range plasmids. Without adequate well-annotated training samples, learning models can fail to extract discriminative feature representations for plasmid host prediction.

**Results:**

To address this problem, we propose a self-correction multi-label learning model called MOSTPLAS. We design a pseudo label learning algorithm and a self-correction asymmetric loss to facilitate the training of multi-label learning model with samples containing some unknown missing labels. We conducted a series of experiments on the NCBI RefSeq plasmid database, the PLSDB 2025 database, plasmids with experimentally determined host labels, the Hi-C dataset, and the DoriC dataset. The benchmark results against other plasmid host range prediction tools demonstrated that MOSTPLAS recognized more host labels while keeping a high precision.

**Availability and implementation:**

MOSTPLAS is implemented with Python, which can be downloaded at https://github.com/wzou96/MOSTPLAS. All relevant data we used in the experiments can be found at https://zenodo.org/doi/10.5281/zenodo.14708999.

## 1 Introduction

Plasmid is a small circular, double-stranded, and independently replicated DNA molecule within a cell (Shintani *et al.* 2015). It is mostly found in bacteria and physically separated from chromosomal DNA. In some cases, the genes encoded by plasmids provide bacteria with beneficial traits such as antibiotic resistance, metal resistance, and virulence catabolic capacity (Svara and Rankin 2011, Li *et al.* 2015). To obtain comprehensive understanding of how plasmids facilitate the evolution of bacteria, we first need to explore the relations between plasmids and their host bacteria.

The host range of a plasmid refers to the microorganisms where a plasmid can transfer, replicate, or persist (Van der Auwera *et al.* 2009, Jain and Srivastava 2013, Popowska and Krawczyk-Balska 2013). Accordingly, plasmids can be classified into narrow-host-range plasmids and broad-host-range (BHR) plasmids. BHR plasmids refer to a group of plasmids that can replicate and stably maintain among organisms of different phylogenetic groups (Jain and Srivastava 2013). For example, plasmid pMOL98 could transfer to and replicate in members of the *α*-, *β*-, and *γ*-proteobacteria (Van der Auwera *et al.* 2009). BHR IncP plasmids are known to spread genes in all gram-negative bacteria (Popowska and Krawczyk-Balska 2013).

BHR plasmids are closely associated with horizontal gene transfer among bacteria of different taxonomic groups. Correctly predicting the host range of BHR plasmids is beneficial for us to explore how plasmids are devoted to the spread of antimicrobial resistance genes among human pathogens (Heuer and Smalla 2012). In addition, with the discovery of more BHR plasmids, their replicons can be used to create recombinant vectors that can replicate and express proteins in different bacteria (Lale *et al.* 2011).

Existing tools designed for plasmid host range prediction can be roughly divided into alignment-based (Robertson *et al.* 2020, Redondo-Salvo *et al.* 2021) and learning-based (Aytan-Aktug *et al.* 2022, Ji *et al.* 2023). The assumption behind alignment-based tools is that plasmids with high sequence similarity tend to share the same host range. Learning-based plasmid host range prediction tools first extract sequence features of plasmids, such as *k*-mer frequency, GC content, codon usage information, replicon type, mobilization (MOB) type, etc. Then, these features are fed into learning models such as convolutional neural network (CNN), transformer, random forest to predict the host range of input sequence. Compared with alignment-based tools, learning-based tools have higher sensitivity for predicting the host range of plasmids that can transfer to distantly related hosts and novel plasmids, particularly those lacking significant alignments with curated plasmids in the reference databases (Aytan-Aktug *et al.* 2022, Ji *et al.* 2023).

Although learning-based tools have superiority on identifying the host range of newly discovered plasmids, there are still some unsolved limitations. Previous research and experiments (Van der Auwera *et al.* 2009, Popowska and Krawczyk-Balska 2013) have demonstrated the existence of BHR plasmids that have multiple host labels among different phylogenetic groups. However, there are no available databases providing detailed and complete host ranges of BHR plasmids. The complete RefSeq plasmid sequences in the NCBI database are solely annotated with the host label from which they are isolated. This challenge can be formulated as a multi-label learning with missing label problem. Compared to single-label learning, multi-label learning aims to identify all relevant annotations for a given sample (Ridnik *et al.* 2021). For instance, a scene may contain multiple objects, computed tomography scans can reveal multiple abnormalities, and satellite images may display multiple landforms. In the field of semi-supervised learning, many pseudo labelling algorithms (Hu *et al.* 2021, Pham *et al.* 2021) have been introduced to mitigate the limitation caused by lacking abundant training samples with complete annotations. Pseudo labels are additional annotations on training samples generated through approaches such as consistency regularization, label propagation, entropy minimization, and so on (Hu *et al.* 2021). These pseudo labels enable the effective utilization of numerous partially labelled or unlabelled samples that would otherwise be discarded. The enlarged size of training samples helps deep learning models extract refined feature representations and further obtain remarkable performance gains on downstream classification or segmentation tasks.

Another challenge of applying multi-label learning models on plasmid host range prediction lies in the highly imbalanced host labels associated with each plasmid. The number of non-host bacteria significantly exceed the number of actual host organisms. This inherent bias can lead to an excessive emphasis on negative labels during model parameters updating, but the contribution of rare positive labels is overwhelmed. And thus, the models make little effort to recognize the similarity between plasmid sequences with the same host labels. In the field of computer vision, many algorithms have been proposed to deal with these problems. Among them, Lin *et al.* (2017) proposed Focal Loss, which reduced the weights of those easily classified negative labels with low predicted probabilities. On the basis of Focal Loss, Ridnik *et al.* (2021) proposed Asymmetric Loss, which omitted the loss of easy negative samples and assigned different focus parameters for positive and negative labels to adjust their weights during model parameter updating. Borrowing from the idea of these successful attempts, to enhance the performance of learning-based tools on plasmid host prediction, we need to mine the missing host labels of training samples and design an appropriate training strategy.

In this work, we propose a self-correction multi-label learning model named MOSTPLAS for genus-level plasmid host range prediction. To the best of our knowledge, this is the first attempt to implement a multi-label learning model for plasmid host range prediction. Restricted by the data availability, there are not enough plasmid sequences with multiple species-level host labels for model training and evaluation. We choose to perform multi-label host prediction at the genus level as it offers a practical and optimal resolution, given the data limitation.

In MOSTPLAS, we design a pseudo label generation algorithm to assign multiple host labels with high confidence to plasmid sequences in the training set. By incorporating pseudo host labels, multi-label learning models can effectively utilize these additional clues to refine the extracted feature representations for different host genera. We also introduce a self-correction asymmetric loss to replace commonly used binary cross-entropy loss, for the training of multi-label learning models. This loss function balances the contribution of negative and positive labels within the training process and enables the models to adaptively recognize missing labels.

We performed a series of experiments on multi-host plasmid test set generated from the NCBI RefSeq database and PLSDB 2025 database, real-world plasmid sequences with experimentally determined host range, and metagenomic data. We compared the performance of MOSTPLAS with existing plasmid host range prediction tools. Experimental results show that MOSTPLAS increases the number of retrieved host labels while keeping a high precision on plasmids with multiple genus-level host labels. On multi-host RefSeq plasmid test set, when all the tools achieved a precision higher than 80%, the recall and F1-score of MOSTPLAS improved by 5.7% and 5.0%, comparing with the second-best tool.

## 2 Materials and method

### 2.1 Overview

**Figure 1.**
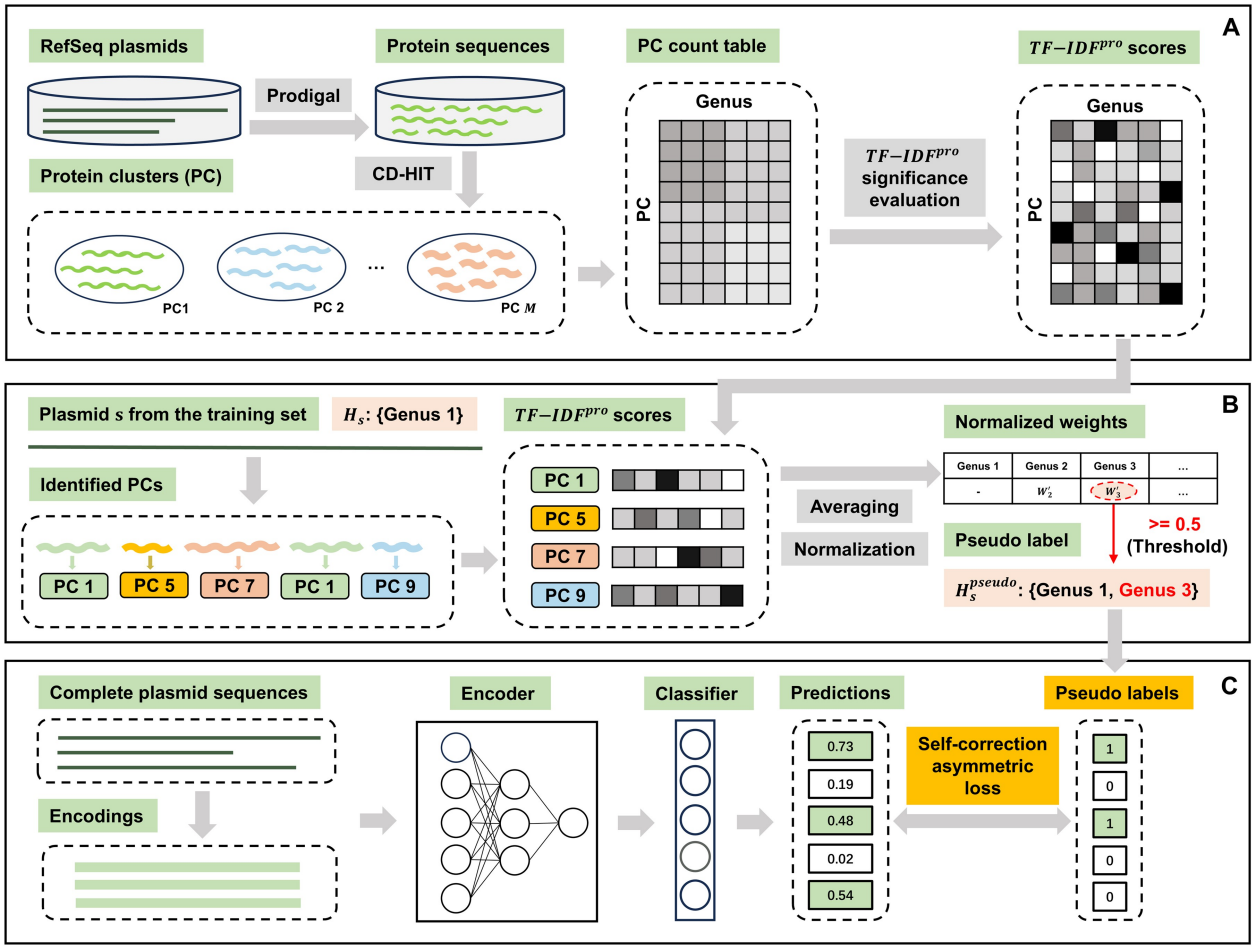
Pipeline of our work. (A) Exploration on the distribution of plasmid-encoded proteins in different host genera. (B) Pseudo label generation process. (C) Model architecture of MOSTPLAS. In the PC count table and ${\text{TF-IDF}}^{\mathrm{pro}}$ score matrix shown in sub figure (A), darker colour represents higher value. Once we obtained the PC significance scores matrix, we identified the corresponding scores of the protein sequences encoded in each plasmid. Based on protein organization, we assigned additional pseudo labels for the plasmids in the training set. The generated pseudo labels were then incorporated together with self-correction asymmetric loss to facilitate the training of multi-label learning models.

MOSTPLAS takes complete plasmid sequences as input and predicts all the possible host labels at genus level in an end-to-end manner. Figure 1C sketches the architecture of the main components of MOSTPLAS. MOSTPLAS first extracts feature representations of the input plasmid sequences using an encoder, such as neural network (NN) or CNN. The obtained feature representations are further passed through the multi-label classifier to predict the possibilities belonging to different genera. The implementation details of the encoder and classifier are presented in Supplementary Section S1. During model training stage, we employed the reliable pseudo labels of the training samples and self-correction asymmetric loss to supervise the parameter updating of encoder and classifier. After training, given a testing plasmid sequence, host genera with probability higher than the defined threshold are selected as the predictions of MOSTPLAS.

In MOSTPLAS, to deal with the challenge of obtaining a substantial number of well-annotated plasmid sequences for the training of multi-label learning models, we design a pseudo host label generation algorithm. We assign extra credible host labels for the plasmid sequences in the training set. With these additional supervision information, encoder can refine the extracted feature representations. With the insightful feature representations containing more characteristics of their hosts, learning models could recognize more host labels for plasmids with high precision. In addition, to cope with another challenge referring to the significant imbalance between the number of positive and negative labels in the training samples, we design a self-correction asymmetric loss. Comparing with the loss function adopted by existing tools, self-correction asymmetric loss highlights the contribution of positive labels and down-weights impact of easy negative labels. Self-correction asymmetric loss also enables the learning models to adaptively recognize the missing positive labels. In the following sections, we will elaborate the details of these two mechanisms.

### 2.2 Pseudo label generation algorithm

Previous research discovers that plasmids with high sequence similarity tend to share the same host range (Redondo-Salvo *et al.* 2020). Experiments find that some plasmids have multiple origins of replication, and these origins are activated separately in different types of hosts (Dewan and Uecker 2023). Survey on transposable elements also concludes that transposons carried by plasmids encode machinery to promote their own mobilization and propagation across their hosts (Jangam *et al.* 2017). These biological characteristics suggest that we can design a pseudo label generation algorithm by mining some useful information hidden in the distribution of plasmid-encoded proteins among different genera. As the host adaptation is largely related to some specific encoded proteins in plasmids, we could identify potential extra hosts based on the protein similarity and organization accordingly. The pipeline is sketched in Fig. 1.

#### 2.2.1 Plasmid-encoded proteins and their host genera

To analyse correlations between a group of plasmid-encoded proteins and specific host genera, we first need to obtain the distribution of plasmid-encoded protein sequences. Specifically, we downloaded all the plasmid sequences from the NCBI RefSeq database. The latest version was released on 9 March 2024 and contained 54 794 sequences. We selected the sequences with assembly level as complete genome and downloaded their corresponding host lineages. As the focus of this work is on bacterial hosts, we removed the sequence with non-bacterial hosts. We also discarded the genera that contain <10 sequences as the number of samples is not sufficient for model training. As a result, we obtained 41 074 complete plasmid sequences corresponding to 216 genera in total.

To obtain the protein organization of each plasmid, we adopted Prodigal (Hyatt *et al.* 2010) for gene prediction and translation of RefSeq plasmid sequences. Then, we applied CD-HIT (Fu *et al.* 2012) (with a threshold of 0.9) to cluster all the protein sequences. After obtaining the protein cluster (PC), we calculated the number of protein sequences corresponding to different genera within each PC and obtained a PC count table as shown in Fig. 1A. This PC count table was used to compute the significance score of a PC to a host genus. With the significance score matrix, we derived the extra pseudo host labels of a plasmid sequence from the training set according to the corresponding significance scores of encoded proteins in this plasmid. In the next subsection, we describe how we compute the significance of a PC to a host genus.

#### 2.2.2 PC significance evaluation

In information retrieval, term frequency-inverse document frequency (TF-IDF) is a commonly used measurement of the importance of a word in a collection of different kinds of documents (Christian *et al.* 2016). We can adopt TF-IDF to evaluate the significance of one PC to a genus, i.e. the PCs generated by CD-HIT can be regarded as the words, and all the genera can be considered as different types of documents. The formulation is given below:

(1) $${\text{TF-IDF}}_{i,j}=\frac{\left|P_{i,j}\right|}{\left|P_{j}\right|}\text{log}\;\frac{\left|G\right|}{\left|G_{i}\right|},$$

where $\left|P_{i,j}\right|$ is the number of times PC *i* appears in genus *j*, $\left|P_{j}\right|$ is the total number of PCs in genus *j*, |G| is the total number of genera, and $\left|G_{i}\right|$ is the number of genera contained in PC *i*. If PC *i* appears more frequently than other PCs in genus *j*, PC *i* has a higher significance to this genus *j*. On the contrary, if PC *i* contains protein sequences belonging to most of the genera, PC *i* has a low TF-IDF score.

However, there are still some limitations on applying TF-IDF to calculate the significance of one PC to a genus. Essentially, TF-IDF only considers the appearance frequency of a PC within each genera, but neglects its appearance frequency among different genera. As a result, it may not accurately reflect the importance of small PC to a genus. For example, if a small PC *i* appears more frequently in genus *j* than other genera, it intuitively should have a higher significance score to genus *j*. However, as size of PC *i* is significantly small than other PCs in genus *j*, it will be assigned with a low significance scores than those large PCs. To incorporate the normalized frequency of a PC in one genus and its relative frequency across different genera, we design TF-IDF^*pro*^ to evaluate the significance of a PC *i* to a genus *j*:

(2) $${\text{TF-IDF}}_{ij}^{\mathrm{pro}}=\sqrt{\frac{\left|P_{i,j}\right|}{\left|P_{.j}\right|}}\sqrt{\frac{\left|P_{i,j}\right|}{\left|P_{i.}\right|}}(\text{log}\;\frac{|G|+1}{|G_{i}|+1}+1),$$

where $|P_{.j}|=\sum_{i}P_{i,j}$, denotes the total number of PCs in genus *j*. $|P_{i.}|=\sum_{j}P_{i,j}$, denotes the total number of times genus *j* oriented protein sequences appear in PC *i*.

#### 2.2.3 Assessment on the TF-IDF^*pro*^ significance scores

**Figure 2.**
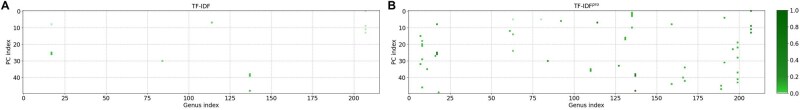
Comparison between the TF-IDF and TF-IDF^*pro*^ significance scores of the smallest 50 PCs. The PCs are sorted by the total number of protein sequences in each PC in descending order. For each cell of the heatmaps, darker colour indicates higher significance score of PC *i* towards genus *j*.

To visualize the difference between TF-IDF and TF-IDF^*pro*^, we first sorted the PCs based on the total number of protein sequences in each PC in descending order. Then, we used heat maps to present the TF-IDF and TF-IDF^*pro*^ significance scores of the smallest 50 PCs in Fig. 2. In each heat map, darker colour represents higher significance scores. While TF-IDF largely ignores the significance of some genus-specific PCs, TF-IDF^*pro*^ can distinguish their contributions.

Compared with TF-IDF, TF-IDF^*pro*^ differentiates the significance scores of small PC to different genera more clearly. Almost all the TF-IDF scores shown in Fig. 2A are zero because they appear less frequently than other large PCs in each genus. In contrast, TF-IDF^*pro*^ assigns higher significance scores for some PCs if their frequencies in one genus are higher than frequencies in other genera. Considering that we generate pseudo labels based on the significance scores of encoded proteins within each plasmid, applying TF-IDF to plasmids that encode a large number of proteins corresponding to small PCs may lead to a significant number of false predictions. Therefore, TF-IDF^*pro*^ is more suitable for pseudo label generation.

**Figure 3.**
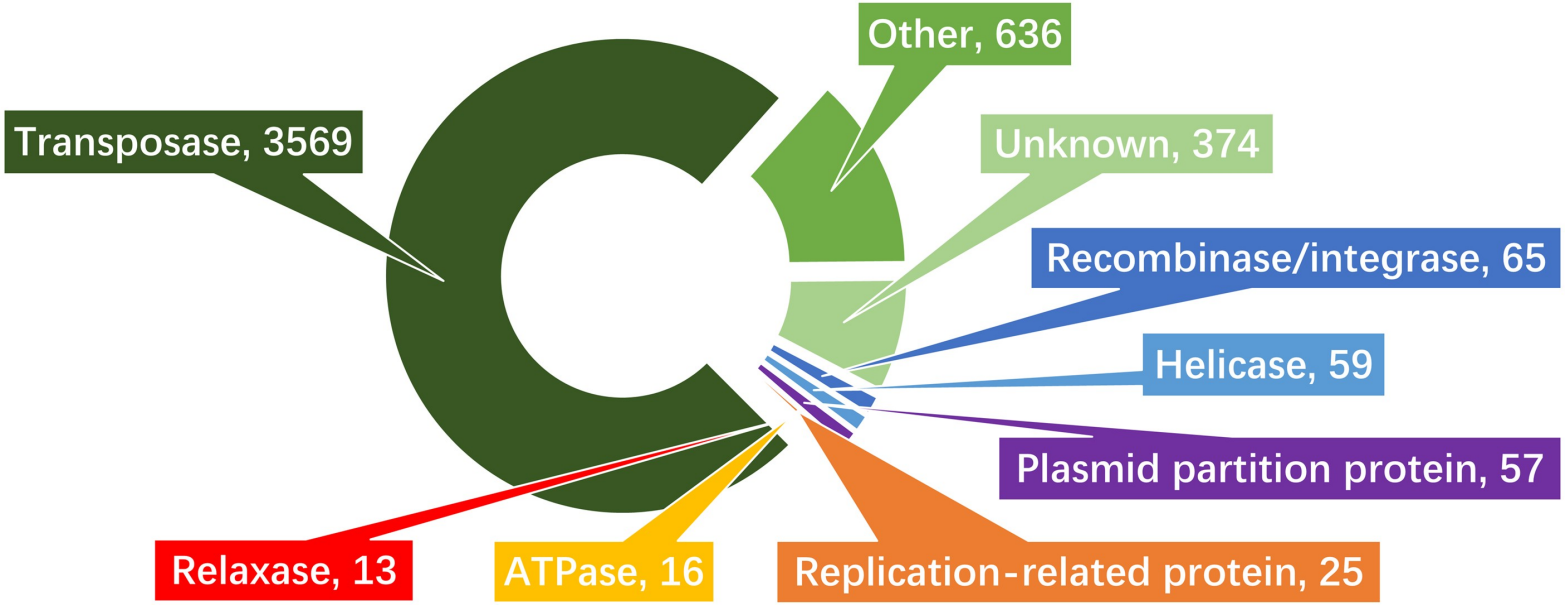
Function annotations of the protein sequences involved in the top 200 PCs with the highest variance towards the TF-IDF^*pro*^ scores of different genera.

We further explored the function of the protein sequences based on TF-IDF^*pro*^ significance scores. Specifically, we summarized the function annotations of the top 200 PCs with the largest variance of TF-IDF^*pro*^ w.r.t. different genera, suggesting that these PCs have different importance to different host genera. The results are shown in Fig. 3.

Among all the selected protein sequences, about three-quarters are transposases, which are a class of enzymes that are capable of binding to the end of a transposon and catalysing their movement across individual hosts (Jangam *et al.* 2017). Research on adaptive process of plasmids (Wedel *et al.* 2023) reveals that the cost of evolved plasmids is compensated for by the acquisition of a putative postsegregational killing system encoded in a transposon. The reduced burden leads to an expansion of the host range of the plasmids (Wedel *et al.* 2023). In addition, ATPase and relaxase are related to plasmid conjugation, helicase participates in plasmid replication process (Thomas *et al.* 2017). Apart from 7.8% proteins with unknown function, the total number of core plasmid genes occupies ∼80% in all selected protein sequences, which demonstrates that TF-IDF^*pro*^ can distinguish the importance of different PCs efficiently.

#### 2.2.4 Assignment of pseudo labels

Once we use the TF-IDF^*pro*^ to compute the importance of a PC to a genus, we use this information to assign possible host labels for plasmid sequences in the training set. With an input plasmid sequence *s*, we first identify the corresponding PCs of all encoded proteins in *s*. The set of all PCs is denoted as $P_{s}$. Then, the overall weight of all the PCs in $P_{s}$ to a genus *j*, i.e. $\bar{W_{j}}$ can be computed by averaging the TF-IDF^*pro*^ significance score of each PC to genus *j*. The equation is given below.

(3) $$\bar{W_{j}}=\frac{1}{\left|P_{s}\right|}\sum_{i\in P_{s}}T{F-\text{IDF}}_{ij}^{\mathrm{pro}},$$

where $\left|P_{s}\right|$ is the total number of encoded protein sequences in the set $P_{s},\;i\in P_{s}$ suggests that we only sum up the TF-IDF^*pro*^ scores of PCs in plasmid sequence *s*. With the averaged weights, we compute the normalized weights $W_{j}^{\prime }$ for all the genera as:

(4) $$W_{j}^{\prime }=\frac{\bar{W_{j}}}{\sum_{j\neq H_{s}}\bar{W_{j}}},$$

where *H_s_* is the genus-level host label of input plasmid *s*, $j\neq H_{s}$ indicates that we sum up the average weights of all the genera except the host label. If the normalized weight of one genus is greater than a threshold, this genus is considered as an extra pseudo label for plasmid sequence *s*. The assignment of pseudo labels is formulated as:

(5) $$H_{s}^{\text{pseudo}}=\{\begin{array}{cc} \{H_{s},j\},\text{if}\;W_{j}^{\prime }\geq \text{threshold} & \\ H_{s},\text{else} & \end{array}.$$

The default threshold is set to 0.5, indicating that each plasmid can have at most one pseudo label. By setting this stringent cutoff, we intend to ensure the high precision and reliability of generated pseudo labels. We explored the impact of different thresholds for pseudo label assignment in Supplementary Section S6. We should note that the proposed pseudo label generation algorithm is only applied for plasmid sequences in the training set. These generated pseudo labels are adopted as the supporting materials for the training of multi-label learning models. In the experiment section, we will evaluate the effect of our pseudo label generation algorithm.

### 2.3 Self-correction asymmetric loss

Traditional binary cross-entropy loss adopted for the training of multi-label learning models equally considers the contribution of positive labels and negative labels. Different from binary cross-entropy loss, self-correction asymmetric loss takes advantage of the loss of positive labels to dominate model parameters updating process. Simultaneously, self-correction asymmetric loss down-weights the contribution of easy samples during model training. Easy samples refer to the negative labels having predicted possibility closer to 0 and positive labels having predicted possibility closer to 1.

(6) $$\begin{array}{cc} & L=-\frac{1}{N}\sum_{i=1}^{N}y_{i}L_{i}^{+}+(1-y_{i})L_{i}^{-}\\ & L^{+}={\left(1-p_{i}\right)}^{\gamma_{+}}\;\text{log}(p_{i})\\ & L^{-}=p_{i}^{\gamma_{-}}\;\text{log}(1-p_{i}) \end{array},$$

where *N* is the total number of genera, *y_i_* is the binary label of input plasmid corresponding to genus *i*, and *p_i_* is the model prediction of input plasmid corresponding to genus *i*. As $p_{i}\in [0,1]$, we set $\gamma_{+}<\gamma_{-}$ so that the weight of negative labels is smaller than that of positive labels.

In our sorted complete plasmid sequences, the number of negative labels is far more than that of positive labels. The trained model is conserved to predict a large value of possibility that the input plasmid sequence belongs to one genus. Therefore, after adequate warming-up training epoch, if the model predicts a large possibility for a negative label, we consider it as a missing positive label. We design a self-correction mechanism to facilitate the training of multi-label learning models under the scenario that some of the host labels of input plasmid sequence are possibly missing.

(7) $$L^{-}=I(p_{i}<\tau )L^{-}(p_{i})+(1-I(p_{i}<\tau ))L^{+}(p_{i}),$$

where *τ* is the threshold to identify a missing label, I(·)∈{0,1} denotes the indicator function, if the condition holds, the value is 1, otherwise the value is 0. We evaluated the influence of different thresholds for identification of missing labels in Supplementary Section S7. We should notice that the self-correction mechanism is performed after several training epochs, which we regard the model has a certain discriminating ability at that moment. We provide a detailed formulation of self-correction asymmetric loss and model training configuration in Supplementary Sections S2 and S3.

## 3 Experiments

In MOSTPLAS, the quality of pseudo labels influences its performance on recognizing the host labels of input plasmid sequences. We first accessed the reliability of the generated pseudo host labels using the multi-host RefSeq plasmid test set. The pseudo labels generated with TF-IDF^*pro*^ scores exhibited significantly higher precision compared to those generated with TF-IDF. The detailed results can be found in Supplementary Section S4. Then, we conducted an ablation study to investigated the contribution of pseudo label generation algorithm and self-correction asymmetric loss. We also compared the performance of MOSTPLAS with other tools across multiple datasets. Since MOSTPLAS focuses on the multi-label classification task, where the number of host labels varies among different plasmid sequences, we employed macro-recall, macro-precision, and F1-score as evaluation metrics. Their detailed formulations are provided in Supplementary Section S5. In addition, we compared the running time of MOSTPLAS with other tools. Among the four tools, MOSTPLAS achieved the fastest speed, which indicated its high computation efficiency for plasmid host range prediction. The detailed results can be found in Supplementary Section S9. Finally, we explored some biological characteristics of plasmids with multiple genus-level host labels.

### 3.1 Dataset

#### 3.1.1 Multi-host RefSeq plasmid test set

As the NCBI database only provides the bacterium from which each plasmid sequence is isolated, we cannot directly obtain the complete host label set for each sequence. To screen possible plasmid sequences with multiple genus-level host labels, we identify near-identical plasmid sequences with different genus-level host labels and combine their hosts as multi-host labels. Specifically, we employed BLASTn (Camacho *et al.* 2009) to perform all-against-all alignments. Aligned sequences with both the identity and coverage above 99% are regarded as near identical. If the genus-level host labels of the two plasmid sequences are different, we consider these two sequences as a cross-genus plasmid sequence with a minimal number of mutations. And thus, they share the combined genus-level host labels, which are the union set of their respective host labels.

We obtained 1391 plasmid sequences and their genus-level multi-host labels. Among them, we removed 63 sequences with only one encoded gene and kept the other 1328 sequences to form the multi-host plasmid test set. Apart from these plasmid sequences with multiple genus-level host labels, the remaining 39 297 plasmid sequences with more than one encoded gene constitute the training set. The detailed host range information of each sequence can be found at the availability and implementation section of abstract.

#### 3.1.2 Multi-host PLSDB plasmid test set

PLSDB 2025 (Molano *et al.* 2025) is a database that provides a curated source of plasmids from the NCBI and INSDC, free from chromosomal and redundant sequences. In the latest version released on 31 May 2024, the database includes 72 360 plasmid sequences. We selected the sequences corresponding to the same 216 genera as the multi-host RefSeq plasmid test set. Similar as multi-host RefSeq plasmid test set, we used BLASTn to recognize plasmid sequences with more than one host label. We also discarded the plasmid sequences with only one encoded gene. As a result, we obtained 3323 plasmid sequences with multiple genus-level host labels and the remaining 64 788 sequences formed the training set. The detailed host range information of each sequence is presented in the availability and implementation section of abstract.

#### 3.1.3 Mob-suite dataset

The authors of MOB-suite (Robertson and Nash 2018) performed a search of publications associated with plasmid accession numbers and manually identified and extracted the host range information of plasmid sequence referred in each article. As the host range of plasmid is experimentally determined and reported in literatures, we adopt this database to evaluate the performance of MOSTPLAS on real data. In our experiment, we chose the sequences classified into wide host range and broad host range groups and removed the sequences with missing host range rank or host range. The detailed host range information of the sorted 92 plasmid sequences is provided in the availability and implementation section of abstract.

#### 3.1.4 Hi-C dataset

Hi-C sequencing can be used to capture three-dimensional genomic interactions between DNA molecules originating in the same cell, and thus is able to link plasmids to their bacterial hosts (Burton *et al.* 2014). With metagenomic Hi-C data collected from wastewater, Stalder *et al.* (2019) linked plasmid contigs to their host chromosomes and obtained 1307 groups of binned contigs. To identify plasmid contigs from all groups of binned contigs, we adopted MOB-recon (Robertson and Nash 2018) and Platon (Schwengers *et al.* 2020). The intersection of the identified plasmids by the two tools was selected as the Hi-C dataset for experiments. We are aware that the host labels provided by Hi-C datasets are not complete. Still, this dataset is valuable to evaluate the performance of plasmid host prediction tools in metagenomic data. Experiment results show that the Top-10 accuracy of MOSTPLAS was 90.3%, which indicated the potentials of MOSTPLAS on narrowing the query scope for plasmid with unknown host range in metagenomic data. More details of the experiments on the Hi-C dataset can be found in Supplementary Section S11.

#### 3.1.5 DoriC dataset

DoriC was first launched in 2007 as a database of replication origins (Oris) in bacterial genomes (Dong *et al.* 2023). In the latest version DoriC 12.0, the database includes Oris of plasmids. The Ori sequences in the DoriC database are predicted based on Z-curve theory and comparative genomic method. We employ this dataset to explore the biological characteristics of Oris in plasmids with multiple genus-level host labels. Based on the predictions of MOSTPLAS, we discovered that plasmids with multiple genus-level host label tend to have more than one replicon. The detailed settings and results on the DoriC dataset are presented in Supplementary Section S12.

### 3.2 Results on multi-host plasmid test set

#### 3.2.1 Ablation study

We first performed an ablation study to explore the effect of pseudo label generation algorithm and self-correction asymmetric loss. As mentioned in Section 2.1 and Fig. 1, in MOSTPLAS, we designed three kinds of multi-label learning benchmarks with different encoders. All three benchmarks were adopted for this experiment, and we employed the same threshold to select their predictions. To validate the reliability and significance of the experimental results, we calculated the confidence intervals with 95% confidence on the F1-scores of three kinds of benchmarks. The results are shown in Table 1.

**Table 1.** Ablation study results of MOSTPLAS on RefSeq and PLSDB datasets.^a^

	Encoder	RefSeq			PLSDB		
		Recall	Precision	F1-score	Recall	Precision	F1-score
Multi-label learning benchmark	NN	39.514	**94.819**	55.782 *(54.269–56.504)*	36.837	94.745	53.048 *(51.310–53.448)*
	CNN	40.686	86.806	55.404 *(54.723–55.671)*	**39.448**	88.985	**54.663** *(52.785–55.832)*
	NN + CNN	**42.314**	92.775	**58.120** *(51.608–60.766)*	36.858	**95.873**	53.246 *(52.768–53.863)*
Multi-label learning benchmark + pseudo labels	NN	48.857	**89.475**	63.203 *(61.575–63.877)*	41.359	**89.081**	56.490 *(56.079–57.006)*
	CNN	49.743	85.532	62.903 *(61.825–63.395)*	**47.134**	80.607	**59.485** *(58.145–60.912)*
	NN + CNN	**53.714**	86.098	**66.156** *(62.398–67.586)*	44.299	86.787	58.658 *(56.989–59.340)*
Multi-label learning benchmark + pseudo labels + self-correction asymmetric loss (MOSTPLAS)	NN	63.100	**84.475**	**72.174** *(71.050–72.647)*	57.537	**81.717**	67.528 *(66.660–67.814)*
	CNN	59.257	82.325	68.912 *(67.326–69.537)*	58.907	78.835	67.429 *(66.882–68.407)*
	NN + CNN	**63.543**	80.443	71.001 *(70.726–71.396)*	**60.478**	80.306	**68.995** *(67.910–69.784)*

a Multi-label learning benchmark denotes that we incorporate the encoder with a multi-label classifier and train the entire model with common binary cross-entropy loss. We highlighted the best performance of each model in bold. The results with italics form refer to the confidence intervals with 95% confidence.

After incorporating pseudo labels, the recall of three kinds of multi-label learning benchmarks increased by 9.3%, 9.1%, and 11.4%, respectively on the RefSeq dataset. Accordingly, the F1-score improved by 7.4%, 7.5%, and 8.0%. On the PLSDB dataset, the improvement of recall was 6.5%, 7.7%, and 7.4%; the improvement of F1-score was 3.4%, 4.8%, and 5.4%, respectively. These large improvements demonstrated that pseudo labels provided more useful and reliable clues during the training stage of learning models. With the help of extra supervision, the encoders could extract more discriminative feature representations for each host genus. If we further replaced traditional binary cross-entropy loss with self-correction asymmetric loss, all three benchmarks achieved higher recall and F1-score. Among them, on the RefSeq dataset, benchmark framework with NN+CNN encoder obtained the highest recall with 63.5%. Benchmark with NN encoder performed the best on precision and F1-score, with 84.5% and 72.2%, respectively. On the PLSDB dataset, benchmark with NN + CNN encoder achieved the highest recall and F1-score as 60.5% and 69.0%. These results suggested that self-correction asymmetric loss facilitated the training of multi-label learning model and helped recognize more host labels of input plasmid sequences with a small sacrifice of precision.

Upon comparing multi-label learning benchmark and MOSTPLAS, we found that their confidence intervals did not overlap. The lower limit of MOSTPLAS’s confidence interval was greater than the upper limit of multi-label learning benchmark’s confidence interval. These results were consistent across all three benchmark variants with different encoders on both the RefSeq and PLSDB datasets. The 95% confidence intervals demonstrated that the performance improvements achieved through the incorporation of pseudo labels and self-correction asymmetric loss were significant. According to Table 1, MOSTPLAS obtained a comparable performance on recall, precision and F1-score with different kinds of encoders. This result indicated that the performance of MOSTPLAS was robust to different options of encoders. In the following experiments, we chose NN as the default encoder.

#### 3.2.2 Performance comparison with other plasmid host range prediction tools

**Figure 4.**
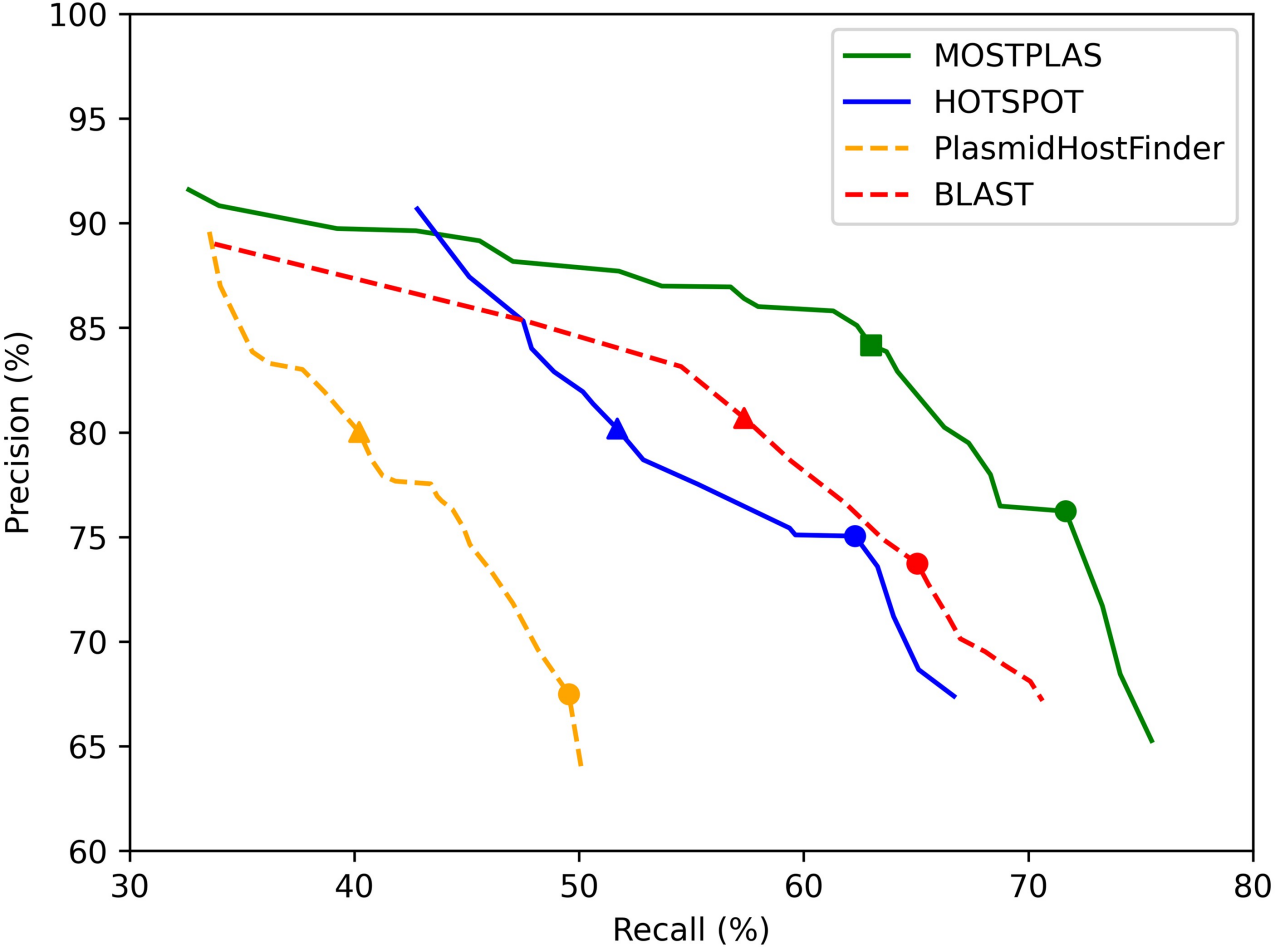
Performance comparison of MOSTPLAS with other plasmid host range prediction tools. • denotes the performance when the tools achieved the highest F1-score. ▲ represents the result when the tools obtained the highest recall with precision above 80%. ■ denotes the performance of MOSTPLAS with default settings.

We benchmarked the performance of MOSTPLAS with available plasmid host prediction tools: BLAST, PlasmidHostFinder (Aytan-Aktug *et al.* 2022), and HOTSPOT (Ji *et al.* 2023). BLAST is a widely used alignment tool with high precision for plasmid host prediction. To obtain the predictions of BLAST, all the plasmid sequences in the multi-host plasmid test set were aligned with the plasmid sequences in the training set of MOSTPLAS. The genus-level host label of the best-matched sequences was regarded as the prediction of the query plasmid. For BLAST, the multi-label predictions were obtained by the top-1 to top-15 alignments based on bitscore ranking. PlasmidHostFinder (Aytan-Aktug *et al.* 2022) and HOTSPOT (Ji *et al.* 2023) are two state-of-the-art learning-based tools. Although these tools are trained in single-label learning manner, they can be modified to function as multi-label learning models for comparison. To achieve this, we employed a threshold to select all eligible predictions instead of choosing the only one predicted genus with the highest probability. To ensure a fair comparison, we utilized different thresholds to obtain the outputs of these two tools. The results are shown in Fig. 4. We also compared the performance of MOSTPLAS with PlasmidHostFinder and HOTSPOT under their default settings. The results are shown in Supplementary Section S8.

Compared with other tools, when the recall of MOSTPLAS improved from 35% to 65%, the decreasing of precision was more gradual. To ensure the reliability of the predictions, we selected a default threshold of 0.4 for MOSTPLAS to determine the host labels of input plasmid sequences. Under the default setting, the recall, precision, and F1-score of MOSTPLAS were 63.0%, 84.2%, and 72.1%, respectively. When BLAST, PlasmidHostFinder, and HOTSPOT attained a similar precision as MOSTPLAS, i.e. the value was above 80%, their highest recall was 57.3%, 40.2%, and 51.7%. The recall of MOSTPLAS was 5.7% higher than that of the second-best tool. When the four tools achieved the highest F1-score, the recall, and precision of MOSTPLAS were both the highest. Compared with the second-best tool (BLAST), the recall, precision, and F1-score increased by 6.6%, 2.5%, and 4.7%, respectively.

The substantial performance improvement achieved by MOSTPLAS highlighted the contribution of pseudo label generation algorithm. Without pseudo labels, plasmids with multiple host genera in the training set were only annotated with one label. During model parameter updating process, these samples only contribute to the feature extraction of one host genus. Consequently, the encoders could only learn coarse feature representations for plasmids with multiple host genera, as the features include partial host label information. In addition, the remarkable enhancement in recall benefits from the self-correction mechanism implemented in the loss function of MOSTPLAS. While pseudo labels provide additional host information for the training samples, there may still be potential host labels that remain undiscovered. The self-correction asymmetric loss enables the model to adaptively recognize these missing positive labels, thereby minimizing the uncertainty arising from the absence of these labels during the decision boundary estimation stage of classifier.

### 3.3 Performance on plasmid sequences with experimentally determined host range

**Figure 5.**
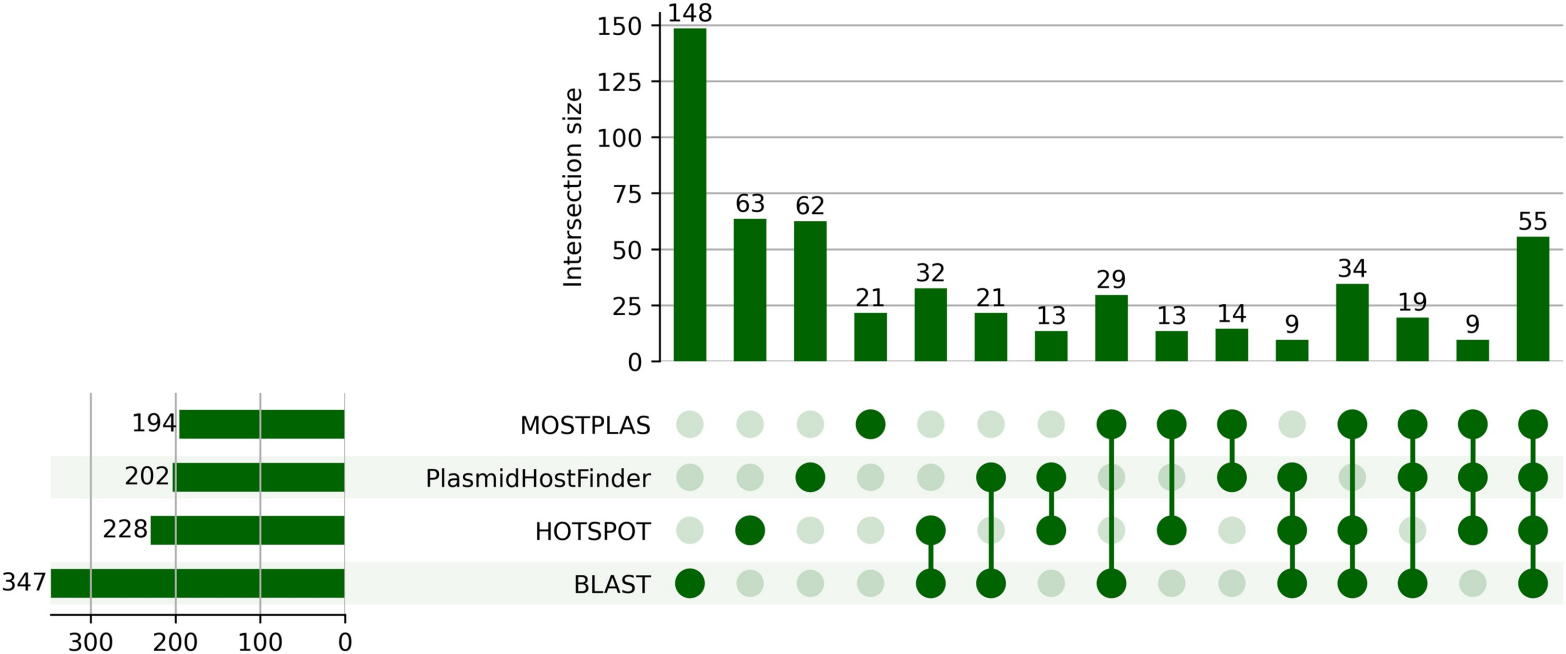
UpSet diagram of the predictions of four plasmid host range prediction tools for plasmid sequences in the Mob-suite dataset.

In the Mob-suite dataset, the host range label of each plasmid is annotated in different taxonomy levels, from superkingdom to family. As MOSTPLAS performs genus-level plasmid host range prediction, we calculated the intersection between its results and different tools to evaluate the performance. We chose the predictions of BLAST, PlasmidHostFinder, and HOTSPOT generated with the thresholds to maximize their F1-scores on multi-host plasmid test set. The prediction comparison of these tools is illustrated in Fig. 5. We also compared the predictions of these tools obtained with the thresholds to achieve a precision higher than 80%. The results are presented in Supplementary Section S10.

MOSTPLAS has the lowest number of predictions, but 89.2% of its predictions overlapped with at least one of the other three tools. HOSTSPOT predicted about 17.5% more host labels than MOSTPLAS, but the number of predictions overlapping with other tools is similar as MOSTPLAS.

These results suggested the predictions of MOSTPLAS on the plasmid sequences of Mob-suite dataset were conserved but reliable. Taking into account both the number of tool predictions and predictions overlapped with at least one other tools, MOSTPLAS outperformed the other tools in terms of the plasmid sequences with experimentally determined host range.

## 4 Conclusion and discussion

In this article, we present a novel self-correction multi-label learning model called MOSTPLAS for genus-level plasmid host range prediction. To the best of our knowledge, this is the first attempt to apply a multi-label learning model to this task. In MOSTPLAS, we design a pseudo label learning algorithms to mitigate the limitation of no available database providing complete host range of BHR plasmid sequences for deep learning models to extract discriminative feature representation for each genus. We also introduce a self-correction asymmetric loss that adjusts the preference of traditional binary cross-entropy loss on the negative labels during model parameters updating. The experiment results on multi-host plasmid test set generated from the NCBI RefSeq database and PLSDB 2025 database, real-world plasmid sequences with experimentally determined host range, and metagenomic data demonstrate the effectiveness of MOSTPLAS.

Although MOSTPLAS showed promising performance under the challenging scenario that the host labels of plasmid sequences include unknown positive labels, there are still areas for improvement in future research. Firstly, MOSTPLAS requires complete plasmid sequences as input, as there is currently insufficient evidence showing the host range relationship between complete BHR plasmid sequences and their corresponding plasmid contigs. Moreover, the host labels of the plasmid sequences in our multi-host plasmid test set were determined with a strict BLASTn alignment threshold. The other predicted non-host bacteria still contain some potential false negative labels. In future work, it would be valuable to investigate the mechanism how the origins of replication, transposons as well as other mobile genes contribute to the host adaption of plasmids function, which may provide us with more clues on determining the host range of plasmid contigs.

## Supplementary Material

btaf075_Supplementary_Data
