# Supplementary material for: MOSTPLAS: a self-correction multi-label learning model for plasmid host range prediction
Source: Bioinformatics. 2025 Feb 17;41(3):btaf075. doi: 10.1093/bioinformatics/btaf075 (PMC11897426; doi:10.1093/bioinformatics/btaf075)
Supplement: btaf075_Supplementary_Data [file btaf075_supplementary_data.zip › Supplementary file.pdf]

Supplementary file for "MOSTPLAS: A Self-correction Multi-label Learning Model for Plasmid Host Range Prediction"

Wei Zou, Yongxin Ji, Jiaojiao Guan and Yanni Sun*

Electrical Engineering Department, City University of Hong Kong, Kowloon, Hong Kong SAR

Content

1. Model architecture 2

2. Formulation of self-correction asymmetric loss 3

3. Training setting 4

4. Evaluation on the reliability of pseudo labels 5

5. Evaluation metric 7

6. Ablation study on the thresholds for the assignment of pseudo labels 8

7. Ablation study on the thresholds for the self-correction mechanism 9

8. Performance comparison with recent plasmid host prediction tools under default settings ...10

9. Running time comparison with recent plasmid host prediction tools 12

10. Results on plasmid sequences with experimentally determined host range 12

11. Results on metagenomic data 13

12. Exploration on the DoriC dataset 14

Reference 16

1. Model architecture

In MOSTPLAS, we designed three different kinds of encoder for feature extraction: Neural Network (NN), Convolutional Neural Network (CNN) and a multi-model integration framework (NN+CNN). These encoders were then cascaded with a classifier together to formed an end-to-end plasmid host range prediction model. In MOSTPLAS, the classifier is implemented by the combination of a fully-connected layer and a sigmoid activation layer. The number of hidden neurons in the fully-connected layer is the same with the number of total genera.

In bioinformatic, k-mer frequency is a widely used feature for feature analysis [1,2]. For NN encoder, we used the 4-mer reverse complementary frequency vector of input plasmid sequence as model input. The dimension of input vector is 136. The encoder is composed of 4 FC layers and the hidden neuron number is set as 2048. 1024, 512 and 256 respectively for each layer. Among two FC layers, we incorporated a BatchNorm1d layer and a ReLU activation layer.

For CNN encoder, we first used Prodigal to predict the start and end site of all encoded genes from input plasmid sequences. Then, we converted the DNA sequences of all encoded gene into 4-mer reverse complementary frequency vectors. At last, we concatenated all the vectors into one matrix according to gene arrangement order and employ the matrix as model input. We restricted the maximum length of the matrix to 200. For plasmid sequences with less than 200 encode genes, we use zero-padding to make sure the input matrix has uniform size as 200×136.

Inspired by the CNN model adopted for text classification [3], the CNN encoder

includes four convolutional layers and each convolutional layer is cascaded with a BatchNorm2d layer and a ReLU activation layer. As the encoder performs convolution operation among contiguous genes for feature extraction, the kernel size of each convolutional layer is set as 2×136, 3×136, 4×136 and 5×136, respectively. The output channel of all convolutional layers is set as 256 and the stride is set to 1. The feature maps output from all convolutional layers are then passed to a global max pooling layer and converted into a vector with dimension as 256. The adopted four vectors are further concatenated together and sent to a FC layer with 256 hidden neurons.

For NN+CNN encoder, we aim to leverage the strengths of both NN and CNN models. The integration of different models can be achieved through feature-level fusion [4] and decision-level fusion [5]. Based on prior research [6], decision-level fusion has demonstrated superior performance compared to feature-level fusion strategies. Therefore, in NN+CNN encoder, we perform decision-level fusion to integrate the output of NN and CNN models, which is formulated as:

$$p_i = \lambda p_i^{NN} + (1 - \lambda) p_i^{CNN}$$
$$L = \lambda L^{NN} + (1 - \lambda) L^{CNN}$$

where p_i^{NN} and p_i^{CNN} are the output of NN model and CNN model, L^{NN} and L^{CNN} are the loss of NN model and CNN model, λ is a hyper-parameter to balance the contribution of two models. In this work, we set λ as 0.6.

2. Formulation of self-correction asymmetric loss

Instead of applying traditional binary cross-entropy loss that is commonly adopted for the training of multi-label learning model, we design a self-correction asymmetric

loss. This loss function pays more attention to the contributions of positive labels and helps the model adaptively recognize the missing positive labels in the training samples. The formulation is shown below:

$$L_{SCAL} = -\frac{1}{N}\sum_{i=1}^{N} y_i L_i^+ + (1 - y_i)L_i^-$$

$$L^+ = (1 - p_i)^{\gamma_+} \log(p_i)$$

$$L^- = \mathbb{I}(p_i < \tau)p_i^{\gamma_-} \log(1 - p_i) + (1 - \mathbb{I}(p_i < \tau))(1 - p_i)^{\gamma_+} \log(p_i)$$

where N is the total number of genera, y_i is the binary label of input plasmid corresponding to genus i, p_i is the model prediction of input plasmid corresponding to genus i. γ_+ and γ_- are parameters to balance the weights of positive and negative labels. τ is the threshold to identify a missing positive label, $\mathbb{I}(\cdot) \in \{0,1\}$ denotes the indicator function, if the condition holds, the value of the function is 1, otherwise the value is 0.

3. Training setting

MOSTPLAS is implemented with Python 3.10.13 and Pytorch 1.12.1 deep learning platform. We also adopt one NVIDIA GeForce RTX 3090 GPU to speed up model training. We use Adam as the optimizer and the initial learning rate is set as 1e-3. The learning rate is decreased to 1/2 of initial learning rate after 50 training epochs and the entire training epoch is set as 100. In self-correction asymmetric loss, the hyperparameters γ_+ and γ_- that adopt to weight the contribution of positive labels and negative labels are set as 1 and 2 respectively. The label correction is performed after 15 epochs and the threshold τ to identify a missing positive label is set as 0.7.

4. Evaluation on the reliability of pseudo labels

A large number of incorrectly labeled samples can severely degrade model performance by introducing noise into the training of multi-label learning models. To evaluate the error rate of generated pseudo labels, we utilized the multi-host RefSeq plasmid test set because this dataset adopts a highly stringent threshold for assigning multi-host labels. Specifically, the host labels of each plasmid sequence were determined by identifying near-identical plasmid sequences using BLASTn. If two plasmid sequences isolated from different organisms exhibited alignments with 99% identity and 99% coverage, we considered them to share the combined genus-level host labels, defined as the union set of their respective host labels. Therefore, the consistency between generated pseudo host labels and BLAST labels obtained by BLASTn alignments can be served as a measurement of the quality of pseudo labels.

In MOSTPLAS, prior to assigning pseudo labels, we first calculated the average TF-IDF^{pro} significance scores for all encoded genes within a plasmid. Then, we performed normalization to obtain the weights of all the genera. In this experiment, we also compare TF-IDF^{pro} and TF-IDF, which calculates the average significance of all encoded genes in a plasmid to derive the weights of all the genera. Then, for each genus, if its weight exceeded the weight corresponding to the host label of the plasmid, it was selected as the pseudo label. We further investigated the impact of normalization process by applying the same normalization process on TF-IDF scores and selected the pseudo labels following TF-IDF^{pro} procedures. The results are shown in Table S1.

Table S1: Performance comparison between different pseudo label generation algorithms.

Method	Percentage of identified BLAST labels (↑)	Error rate (↓)
TF-IDF	35.635	74.122
TF-IDF (normalization)	17.311	44.131
**TF-IDF^{pro}**	**19.982**	**16.699**

In the table, the percentage of identified BLAST labels represents the ratio of the intersection of pseudo labels and BLAST labels to the total number of BLAST labels. The error rate indicates the ratio of inconsistent labels between pseudo labels and BLAST labels to the total number of pseudo labels. It should be noted that the provided error rate is an upper bound on the actual error rate for the multi-host RefSeq plasmid test set. Due to the lack of plasmid dataset with experimentally determined multi-host labels, the BLAST labels were determined using a stringent BLASTn threshold of 99% identity and 99% coverage. Consequently, the BLAST labels may contain some false negatives. The actual host labels for these plasmids can be larger and will not be included under the stringent BLAST cutoffs.

As shown in the results, after normalization, the error rate significantly reduced by 30.0% with the sacrifice of missing 18.3% BLAST labels. When we replaced TF-IDF with TF-IDF^{pro} for measuring the significance of PCs, the error rate further decreased by 27.5%, and the percentage of identified BLAST labels slightly increased to 20%. Under the stringent threshold, the error rate of pseudo labels generated by TF-IDF^{pro} was below 15%, demonstrating its reliability. These experimental results also indicated that the pseudo label generation algorithm adopted in MOSTPLAS was conserved as it tended to make fewer predictions but ensure the high precision of the pseudo labels. Additionally, we investigated some errors in the pseudo labels generated by both TF-IDF and TF-IDF^{pro}.

We selected several examples and presented the analysis results in Table S2.

Table S2: Examples of pseudo labels generated by TF-IDF and our TF-IDF^{pro}. (×) denotes falsely predicted labels.

Sequence id	Isolated organism	BLAST label	Pseudo label generated by TF-IDF	Pseudo label generated by TF-IDF^{pro}
NZ_AP026682	Salmonella	Enterobacter<br>Leclercia	Enterobacter<br>Leclercia<br>Phytobacter **(×)**<br>Serratia **(×)**	Enterobacter
NZ_OW967522	Klebsiella	Enterobacter<br>Phytobacter	Citrobacter **(×)**<br>Enterobacter<br>Leclercia **(×)**<br>Phytobacter<br>Salmonella **(×)**<br>Serratia **(×)**	Enterobacter
NZ_CP115347	Escherichia	Salmonella	Shigella **(×)**	Shigella **(×)**
NZ_CP067254	Escherichia	Klebsiella	Shigella **(×)**	Shigella **(×)**

By comparing the results in the first two rows, we observed that while TF-IDF successfully recognized all the BLAST labels, it also generated a large number of predictions that were not included in the BLAST label set. This observation aligned with the findings presented in Table S1, where both the percentage of identified BLAST labels and the error were notably high. In contrast, the number of pseudo labels generated by TF-IDF^{pro} was relatively small, yet the majority were consistent with BLAST labels. For the last two plasmid sequences, the pseudo labels generated by TF-IDF and TF-IDF^{pro} were identical but incorrect. Upon further investigation, we found that the pseudo labels belonged to the same family as the BLAST labels and the isolated organisms.

5. Evaluation metric

To evaluate the performance of MOSTPLAS, we employ recall, precision, and F1-score as metrics. As the number of plasmids with different amount of genus-level host labels

is imbalanced, we adopted macro-averaging across the results of different genera and reported the mean value to represent the performance on the entire data. The formulations of macro-recall, macro-precision and F1-score are denoted as the following:

$$macro - recall = \frac{1}{N}\sum_{i=1}^{N}\frac{TP_i}{TP_i + FN_i}$$

$$marco - precision = \frac{1}{N}\sum_{i=1}^{N}\frac{TP_i}{TP_i + FP_i}$$

$$F1 - socre = \frac{2 \times marco - precision \times macro - recall}{marco - precision + macro - recall}$$

where N is the total number of genera, TP_i denotes correctly classified host label for genus i, FN_i is the number of missed host label, FP_i represents falsely predicted host label.

6. Ablation study on the thresholds for the assignment of pseudo labels

We examined the impact of varying thresholds on the assignment of pseudo labels. In MOSTPLAS, after obtaining the normalized weights of all the genera with respect to the encoded genes in a plasmid sequence, we employed a default threshold of 0.5 to assign pseudo labels. This threshold is stringent because it ensures that each plasmid sequence in the training set could have at most one pseudo label. To assess the effect of different thresholds, we set the values from 0.3 to 0.7, with interval of 0.1 to generate different pseudo labels. These pseudo labels were then used to train MOSTPLAS, and we evaluated its performance on the multi-host RefSeq plasmid test set. Except for the different pseudo labels, all other settings remained the same. The results of these

experiments are presented in Fig. S1.

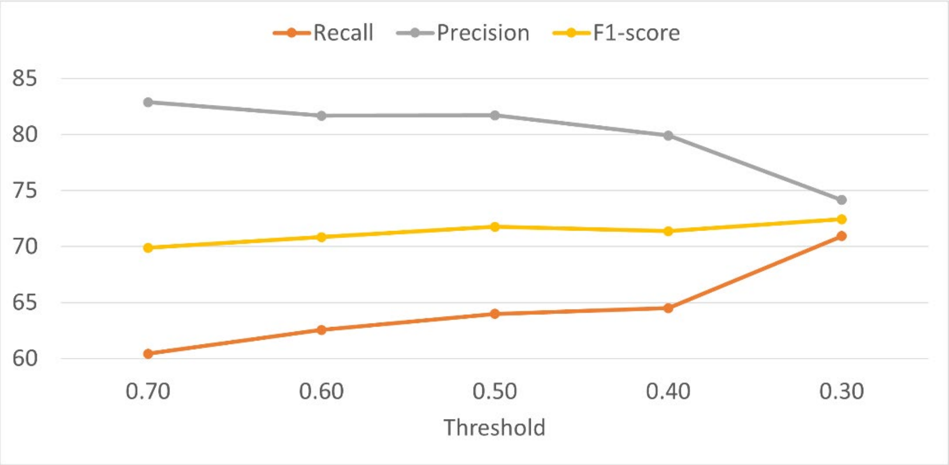

Fig. S1: Performance comparison of MOSTPLAS with different thresholds to assign pseudo labels.

When the threshold decreased from 0.7 to 0.3, the recall increased from 60% to 70%. At the same time, the precision decreased from 83% to 75%. The F1-score remained relatively stable, fluctuating around 70%. These results indicated that lowering the threshold increased the number of generated pseudo labels, leading MOSTPLAS to predict more host genera. Among the five threshold values tested, a threshold of 0.5 enabled MOSTPLAS to achieve the highest recall while maintaining a precision above 80%. Thus, this threshold remains as the default setup in MOSTPLAS.

7. Ablation study on the thresholds for the self-correction mechanism

We also performed an ablation study on the threshold used in the self-correction mechanism. In the self-correction asymmetric loss, after several warming up epochs, if the predicted probability for a negative label exceeds 0.7, it is considered ass a missing positive label. To investigate the effect of different thresholds, we varied the threshold from 0.9 to 0.6, with an interval as 0.05. The experimental results indicated that when the

threshold was set below 0.6, the parameters of MOSTPLAS failed to converge during model training. The results of this study are illustrated in Fig. S2.

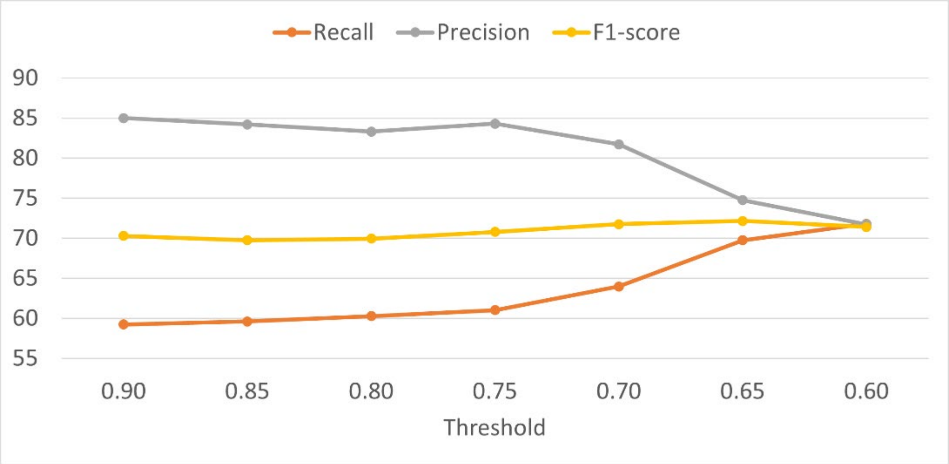

Fig. S2: Performance comparison of MOSTPLAS with different thresholds for self-correction mechanism.

When the threshold was decreased from 0.9 to 0.7, the precision decreased slightly from 85% to 82%, while the recall increased from 59% to 64%. However, further decreasing the threshold from 0.7 to 0.6 resulted in a significant improvement in recall from 64% to 71%, but the precision dropped dramatically. To achieve higher recall while maintaining precision above 80%, we selected a threshold of 0.7 for the self-correction asymmetric loss in the training of MOSTPLAS.

8. Performance comparison with recent plasmid host prediction tools under default settings

We compared the performance of MOSTPLAS with PlasmidHostFinder [7] and HOTSPOT [8] under their default settings. As PlasmidHostFinder and HOTSPOT are both trained in a single-label learning manner, to have a fair comparison, we chose the top-1 prediction of BLAST. The results are shown in Fig. S3.

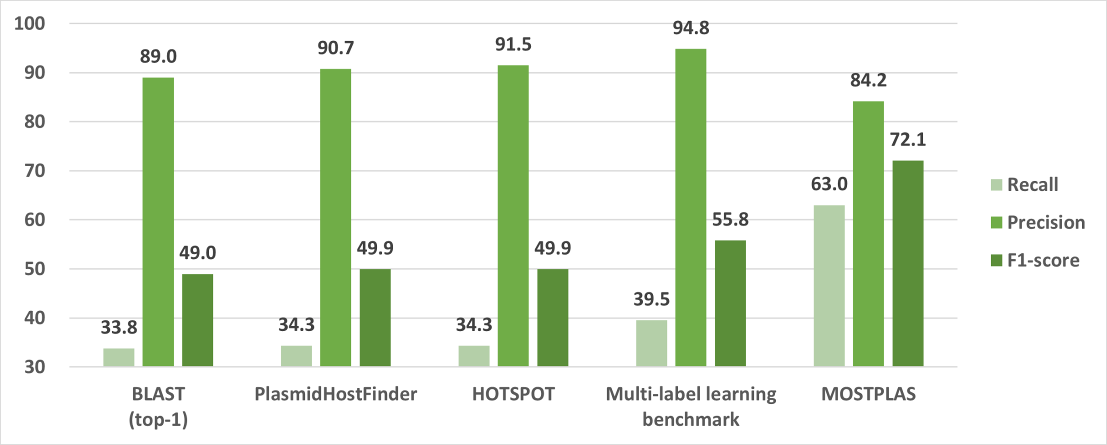

Fig. S3: Performance comparison of MOSTPLAS with single-label learning tools. BLAST (top-1) denotes we chose the genus-level host labels of the top one best matched sequence as the predictions of BLAST.

Under the default setting, PlasmidHostFinder [7] and HOTSPOT [8] obtained a comparable performance with BLAST. Although the precision of these three tools was all above 90%, the recall was all lower than 40%, which leads to the F1-score was around 50%. However, if we compared multi-label learning benchmark with the existing plasmid host range prediction tools, the recall and F1-score increased about 5%, and the precision was basically unchanged. Furthermore, in MOSTPLAS, after incorporating pseudo host labels and self-correction asymmetric loss, the recall dramatically increased to around 65% and accordingly the F1-score was significantly enhanced to over 70%. These results indicated that without the help of pseudo host label generation algorithm and self-correction asymmetric loss, the performance of multi-label learning benchmark is similar as single-label learning models. These two mechanisms boosted the performance of multi-label learning benchmark on recognizing more host labels but without sacrifice of precision to a great extent.

9. Running time comparison with recent plasmid host prediction tools

To evaluate the computation efficiency of MOSTPLAS, we compared its running time with other plasmid host prediction tools. In this experiment, all the tools were tested on four INTEL Xeon X5690 CPUs and one NVIDIA GeForce RTX 3090 GPU. We reported the total running time of predicting the host labels of all 1,328 sequences in the multi-host RefSeq plasmid test set. As MOSTPLAS adopted GPU to speed up the prediction process by default, we also evaluated the running time of MOSTPLAS with only CPU configuration. The results were shown in Table S3.

Table S3: Running time comparison between MOSTPLAS and other plasmid host prediction tools.

Tools	Running Time
BLAST	21 minutes 56 seconds
PlasmidHostFinder [7]	6 hours 44 minutes 9 seconds
HOTSPOT [8]	11 minutes 16 seconds
MOSTPLAS (CPU only)	2 minutes 18 seconds
MOSTPLAS	1 minute 38 seconds

MOSTPLAS achieved the fastest speed to predict the host labels of 1,328 plasmid sequences in the multi-host RefSeq plasmid test set. With only CPU configuration, MOSTPLAS still can save about 90% running time comparing to BLAST (alignment-based tool) and 80% running time comparing to HOTSPOT (learning-based tool). This result indicated the high computation efficiency of MOSTPLAS.

10. Results on plasmid sequences with experimentally determined host range

We calculated the intersection between the predictions of MOSTPLAS and other plasmid host range prediction tools: BLAST, PlasmidHostFinder and HOTSPOT. The predictions of these three tools were obtained with the thresholds that achieving a precision higher than 80%. Under these thresholds, the predictions are considered with

high reliability. The results are shown in Fig. S4.

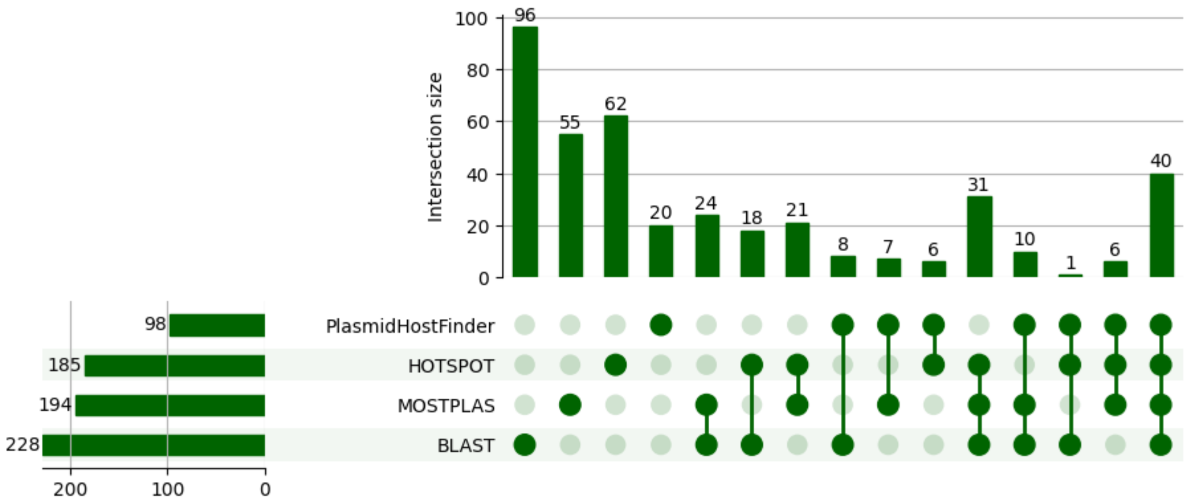

Fig. S4: UpSet diagram of the predictions of four plasmid host range prediction tools for plasmid sequences in the Mob-suit dataset.

Among the four tools, MOSTPLAS predicted the second largest number of host labels, which is similar as HOTSPOT. The number of predictions without overlapping with other tools obtained by PlasmidHostFinder was the smallest and about 40% of MOSTPLAS. However, in MOSTPLAS, the number of predicted host labels overlapped with at least one of the other tools is about two times than that of PlasmidHostFinder.

11. Results on metagenomic data

To evaluate the performance of MOSTPLAS on metagenomic data, we conducted experiments on the Hi-C dataset and compared the results with HOTSPOT. As the identified plasmid contigs in the Hi-C dataset were annotated with single-labels, we adopted accuracy as the evaluation metric. The accuracy is calculated as the number of correctly predicted plasmids divided by the total number of plasmids in the Hi-C dataset. The results are shown in Table S4.

Table S4: Performance comparison on Hi-C dataset. In addition to experiments with the default setting of MOSTPLAS and HOTSPOT, we also compared the Top 3, Top 5 and Top 10 accuracy of MOSTPLAS and HOTSPOT.

Accuracy	MOSTPLAS	HOTSPOT
Default setting (Threshold=0.4)	57.541	/
Default setting (Top 1)	/	50.348
Top 3	53.132	65.429
Top 5	81.206	75.870
Top 10	90.255	79.118

Under the default setting, MOSTPLAS achieved a 7.2% higher accuracy than HOTSOPT. Although the Top3 classification accuracy of MOSTPLAS was lower than that of HOTSPOT, the remaining Top 5 and Top 10 classification accuracy were much higher. The Top 10 accuracy of MOSTPLAS was 90.3%, which indicated that we had high confidence that one of the top ten candidates was the host of input plasmid sequences. This result also demonstrated the effectiveness of MOSTPLAS on narrowing the query scope for plasmid with unknown host range in metagenomic data.

12. Exploration on the DoriC dataset

Previous research concludes that BHR plasmids may contain multiple basic replicons and different replicons will be activated in different hosts, which helps extend the plasmid host range [9]. To examine this characteristic using the identified multi-host plasmids identified by MOSTPLAS, we utilized DoriC12.0 [10], a database that collects several Ori sequences from plasmids. In this experiment, we initially used trained MOSTPLAS to predict the genus-level host labels for all complete plasmid sequences downloaded from the NCBI RefSeq database. Subsequently, we selected the sequences with at least 2 host labels and performed alignments using BLASTN against the Ori sequences in the

DoriC12.0 database. For each alignment of a plasmid sequence, if both the identity and coverage exceeded 95%, we consider this plasmid includes the matched Ori sequence. Overall, we obtain 20,287 sequences with at least 2 host labels, and the distribution of their Ori numbers is illustrated in Fig. S5.

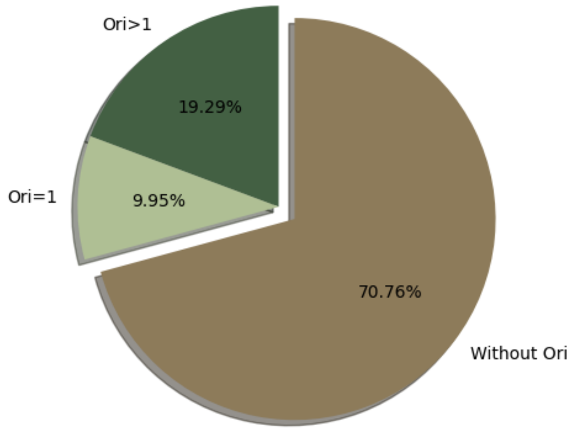

Fig. S5: The distribution of the Ori number in the predicted plasmids with more than two genus-level host labels.

Approximately 70.8% plasmids in our experiment had no alignments with the OriC sequences present in the DoriC12.0 database. This discrepancy can be attributed to the limited coverage of the database, which currently includes only 1,184 Ori sequences extracted from plasmids. Considering the rapid increase in the number of complete plasmid sequences available in the RefSeq database, it is likely that numerous Ori sequences in plasmids remain undiscovered. Additionally, the experiment results reveal that the number of plasmids containing multiple Ori sequences is two times compared to those with a single Ori. This observation suggests that plasmids with multiple genus-level host label tend to have more than one replicon, which is consistent with existing literature [9]. This experiment underscores the importance of identifying Ori sequences within plasmids.

Reference

[1] J. Wen, Y. Liu, Y. Shi, et al., “A classification model for lncRNA and mRNA based on k-mers and a convolutional neural network,” BMC bioinformatics, 2019, 20: 1-14.

[2] H. Yan, A. Bombarely and S. Li, “DeepTE: a computational method for de novo classification of transposons with convolutional neural network,” Bioinformatics, 2020, 36(15): 4269-4275.

[3] S. Wang, M. Huang and Z. Deng, “Densely connected CNN with multi-scale feature attention for text classification,” International Joint Conference on Artificial Intelligence (IJCAI). 2018, 18: 4468-4474.

[4] H. D. Nguyen, S. H. Kim, G. S. Lee, et al., “Facial expression recognition using a temporal ensemble of multi-level convolutional neural networks,” IEEE Transactions on Affective Computing, 2019, 13(1): 226-237.

[5] H. Jung, S. Lee, J. Yim, et al., “Joint fine-tuning in deep neural networks for facial expression recognition,” Proceedings of the IEEE international conference on computer vision (ICCV), 2015: 2983-2991.

[6] Z. Zhao, Q. Liu and S. Wang, “Learning deep global multi-scale and local attention features for facial expression recognition in the wild,” IEEE Transactions on Image Processing, 2021, 30: 6544-6556.

[7] D. Aytan-Aktug, P. T.L.C. Clausen, J. Szarvas, et al., “PlasmidHostFinder: Prediction of Plasmid Hosts Using Random Forest,” mSystems, 2022, 7:e01180-21.

[8] Y. Ji, J. Shang, X. Tang and Y. Sun, “HOTSPOT: hierarchical host prediction for assembled plasmid contigs with transformer,” Bioinformatics, 2023, 39(5):btad283.

[9] N. Hülter, J. Ilhan, T. Wein, et al., “An evolutionary perspective on plasmid lifestyle modes,” Current opinion in microbiology, 2017, 38: 74-80.

[10] M. Dong, H. Luo and F. Gao, “DoriC 12.0: an updated database of replication origins in both complete and draft prokaryotic genomes,” Nucleic Acids Research, 2023, 51(D1): D117-D120.
